# Supplementary material for: Effect of Freeze–Thaw Cycles on the Freshness of Prepackaged Penaeus vannamei
Source: Foods. 2024 Jan 18;13(2):305. doi: 10.3390/foods13020305 (PMC10814677; doi:10.3390/foods13020305)
Supplement: Supplementary file 1 [file foods-13-00305-s001.zip › foods-2800457-supplementary.pdf]

Effect of freeze-thaw cycles on the freshness of prepackaged *Penaeus vannamei*

Table S1 Sensory scoring scale for *Penaeus vannamei*

	Fresh (80~100)	Sub-fresh (50~80)	Spoilage (0~50)
Odours (0.2)	Inherent freshness and sweetness of fresh prawns, no off-flavours	Slightly fishy	Noticeable rotting odour and fishy taste
Textures (0.3)	Tissue is elastic and shiny	Reduced tissue elasticity and dulling of lustre	Tissue loses elasticity and becomes matte in texture
Appearance (0.3)	The carapace is tightly jointed and the body colour is greenish grey	The flesh and shell begin to detach and the head of the shrimp begins to darken and brown.	Flesh and shell can be detached easily, shrimp head is basically off, brain is out, shrimp body is yellowish.
Boiling quality (0.2)	The soup is clear and unadulterated, with a light sweet shrimp flavour	The broth is a little cloudy, a small amount of tissue was shed and a slight odour emerged	The soup is cloudy, the meat is dislodged and suspended, soft and rotten, the odour is more obvious

Table S2 Sensor array and performance of PEN3 electronic nose

Sensor serial number	Sensor name	Sensor performance descriptions
1	W1C	Aromatic components
2	W5S	High sensitivity, sensitive to nitrogen oxides
3	W3C	Ammonia, sensitive to aromatic components
4	W6S	Selective mainly for hydrogen
5	W5C	Alkanes, aromatic components
6	W1S	Sensitive to methane
7	W1W	Sensitive to sulphides
8	W2S	Sensitive to ethanol
9	W2W	Aromatic components, sensitive to organosulphides
10	W3S	Sensitive to alkanes

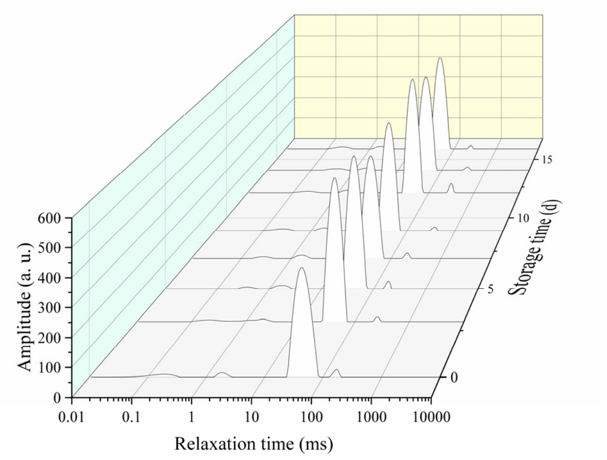

Figure S1. Distribution of relaxation time T_2 of F-T groups.

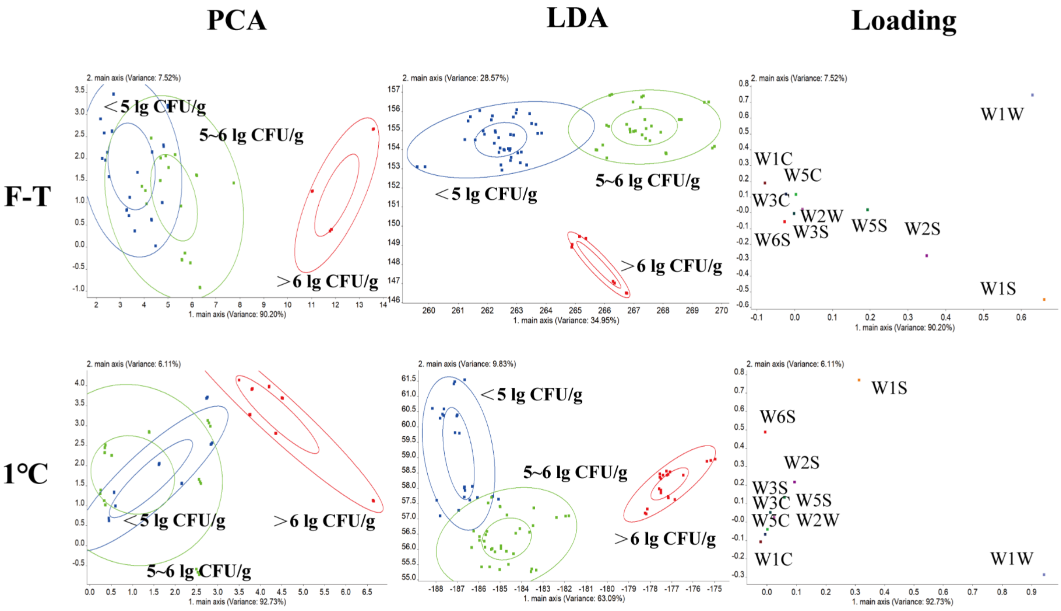

Figure S2. Plot of PCA, LDA and loading analyses for different TPA contents of shrimp.

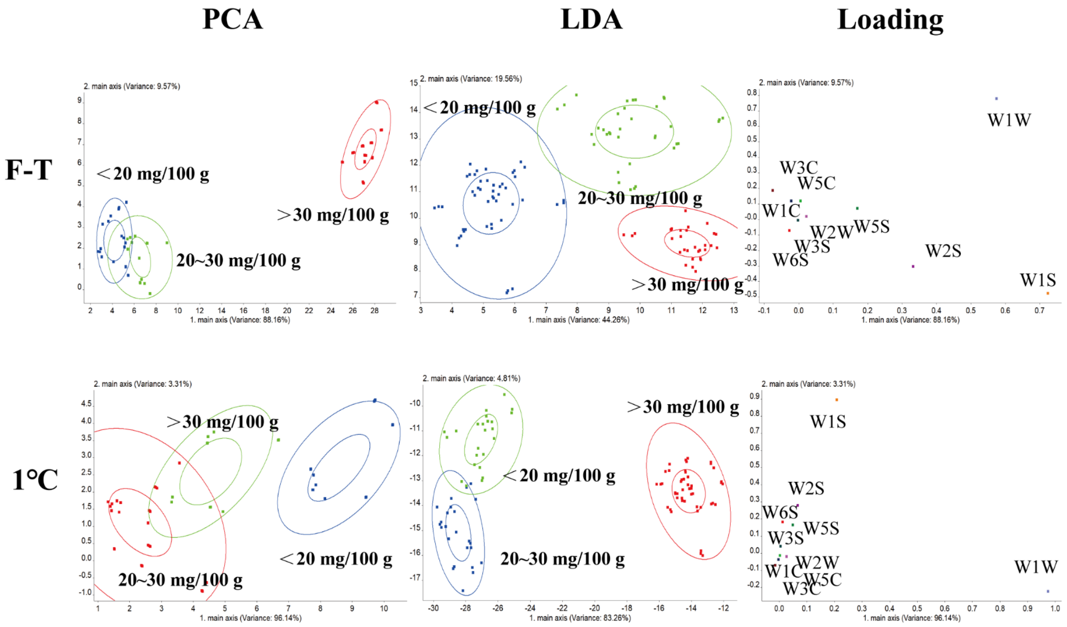

Figure S3. Plot of PCA, LDA and loading analyses for different TVB-N contents of shrimp.
